# Broad-Spectrum Antiviral and Antibacterial Activity of the Scorpion Venom Peptide HP1090

**DOI:** 10.3390/toxins18060268

**Published:** 2026-06-16

**Authors:** Ariel J. Asuzano, Lia-Raluca Olari, Nourice Jaber, Verena Vogel, Marina S. Fam, Armando A. Rodríguez Alfonso, Nico Preising, Ludger Ständker, Barbara Spellerberg, Hans-Georg Breitinger, Ulrike Breitinger, Jan Münch

**Affiliations:** 1 Institute of Molecular Virology, Ulm University Medical Center, 89081 Ulm, Germany; ariel.asuzano@uni-ulm.de (A.J.A.);; 2 Department of Genomics, Medfuture Institute for Biomedical Research, Iuliu Hatieganu University of Medicine and Pharmacy, 400337 Cluj-Napoca, Romania; 3 Institute of Medical Microbiology and Hygiene, Ulm University Medical Center, 89081 Ulm, Germany; 4 Department of Biochemistry, Faculty of Pharmacy and Biotechnology, German University in Cairo, Cairo 11835, Egypt; 5 ULMTeC Core Facility for Functional Peptidomics, Ulm University Medical Center, 89081 Ulm, Germany; 6 ULMTeC Core Facility of Mass Spectrometry and Proteomics, Ulm University Medical Center, 89081 Ulm, Germany

**Keywords:** AMP, HP1090, venom peptide, ESKAPE pathogens, lipid bilayer disruption, membrane permeabilization, selective toxicity

## Abstract

HP1090 is a short, cationic, amphipathic peptide derived from scorpion venom and previously described as a membrane-active antiviral compound. Here, we primarily characterize the antiviral activity of HP1090 and assess whether additional antibacterial effects are consistent with membrane-disruptive properties. Chemically synthesized HP1090 exhibited dose-dependent virucidal activity against multiple enveloped viruses, including herpes simplex virus type 1 and 2 (HSV-1, HSV-2), human immunodeficiency virus type 1 (HIV-1), and Zika virus (ZIKV), with IC_50_ values ranging from 14.7 to 56.1 µg/mL. No activity was observed against the non-enveloped human rhinovirus 14 (HRV14), suggesting strict dependence on a viral lipid envelope. Consistent with a membrane-targeting mechanism, HP1090 induced rapid and concentration-dependent permeabilization of virus-like liposomes. HP1090 also displayed antibacterial activity against selected clinically relevant pathogens in agar-based growth inhibition assays. However, antibacterial effects required substantially higher concentrations (>125 µg/mL) and varied between bacterial species, with some strains showing little or no susceptibility. Membrane permeabilization assays in *Listeria monocytogenes* demonstrated disruption of bacterial membrane integrity as a contributing mechanism. No cytotoxicity was observed on mammalian cell lines at effective antiviral concentrations. Together, these findings establish HP1090 as a membrane-active venom peptide and, by linking envelope-dependent viral inactivation with bacterial membrane permeabilization, support a shared biophysical mode of action relevant to the development of membrane-targeting anti-infectives.

## 1. Introduction

The rapid emergence of antiviral drug resistance and the global spread of multidrug-resistant (MDR) bacterial pathogens represent an escalating threat to public health. Most currently approved antiviral and antibacterial drugs act by targeting specific viral or bacterial enzymes or pathways, such as polymerases, proteases, or cell wall biosynthesis machinery. While these approaches can be highly effective, they are intrinsically vulnerable to resistance development driven by single amino-acid substitutions, genetic recombination, or horizontal gene transfer. Consequently, there is increasing interest in alternative anti-infective strategies that exploit conserved structural features of pathogens and operate through mechanisms that are inherently less prone to resistance evolution [1,2].

Antimicrobial peptides (AMPs) constitute an evolutionarily conserved component of innate immunity and represent a particularly promising class of anti-infective agents. Many AMPs exert their activity through direct interaction with microbial membranes rather than through inhibition of defined protein targets. Because lipid membranes are essential for pathogen viability and infectivity and cannot be readily modified without compromising fitness, membrane-active peptides often display rapid killing kinetics, broad activity spectra, and a reduced propensity for resistance development [1,2].

Lipid membranes represent a shared structural vulnerability across diverse classes of pathogens. Enveloped viruses depend on host-derived lipid bilayers for entry and infectivity, whereas bacterial membranes are enriched in negatively charged phospholipids that distinguish them from mammalian cell membranes. Peptides that selectively target these lipid features can inactivate pathogens through physical membrane disruption, rather than by interfering with intracellular replication or metabolic pathways [2,3,4]. Such a mechanism predicts activity against a broad range of enveloped viruses and bacteria while sparing non-enveloped viruses and host cells, provided sufficient selectivity is achieved [5,6,7].

Venom-derived peptides are a particularly rich source of membrane-active antimicrobial molecules. Scorpion venoms, in particular, contain numerous short, cationic, amphipathic peptides that lack disulfide bonds and typically adopt α-helical conformations in membrane-mimicking environments [8]. These non-disulfide-bridged peptides are well known for their antibacterial and antifungal activities and, more recently, have emerged as a source of antiviral peptides [7]. Within this context, HP1090 represents one of the first scorpion venom-derived peptides with experimentally validated antiviral activity [8].

HP1090 is a short cationic peptide originally identified from the venom gland of the scorpion *Heterometrus petersii* [8]. The peptide consists of 13 amino acids, is C-terminally amidated, and adopts an amphipathic α-helical structure in membrane-like environments. Based on its sequence and structural features, HP1090 belongs to the family of non-disulfide-bridged scorpion venom peptides, which are widely recognized for membrane-disruptive antimicrobial activity [5]. HP1090 was initially described as a potent antiviral peptide active against hepatitis C virus [8], an enveloped RNA virus, with activity attributed to direct disruption of the viral envelope rather than inhibition of intracellular replication.

In addition to its antiviral properties, HP1090 has been reported to exhibit antibacterial activity against both Gram-positive and Gram-negative bacteria [8,9]. However, the initial report by Yan et al. primarily describes this activity without providing corresponding experimental data, and only limited follow-up studies have experimentally validated antibacterial effects, for example against selected *Escherichia coli* strains [9]. Consequently, the antibacterial spectrum of HP1090 has not been systematically investigated, and its activity against clinically relevant pathogens remains poorly defined. Moreover, although membrane disruption has been proposed as a unifying mechanism underlying both its antiviral and antibacterial effects, direct experimental evidence linking these activities remains scarce.

In the present study, we comprehensively characterize the antiviral and antibacterial activity of chemically synthesized HP1090 and test its membrane-disruptive properties using virus-like liposomes and bacterial membrane permeabilization assays. We demonstrate that HP1090 exhibits broad, dose-dependent antiviral activity against multiple enveloped viruses, lacks activity against a non-enveloped virus, and shows no cytotoxicity in mammalian cells at antivirally active concentrations. Furthermore, we show that HP1090 disrupts virus-like lipid bilayers in a rapid and concentration-dependent manner and inhibits the growth of clinically relevant bacterial pathogens through membrane permeabilization. Together, these data establish a unified membrane-disruptive mechanism underlying the dual antiviral and antibacterial activity of HP1090.

## 2. Results

### 2.1. HP1090 Selectively Inhibits Enveloped Viruses Through a Direct Virucidal Mechanism

Prior to functional characterization, the identity and purity of chemically synthesized HP1090 were confirmed by analytical HPLC and mass spectrometry (Supplementary Figures S1–S3). HP1090 is a 13-residue peptide (IFKAIWSGIKSLF) synthesized with a free, non-amidated C-terminus, giving a net positive charge of +2. The helical wheel projection reveals a pronounced amphipathic organization, with hydrophobic and polar residues segregating on opposite faces of the predicted α-helix (Supplementary Figure S1). These structural features are characteristic of membrane-active antimicrobial peptides and are consistent with the proposed ability of HP1090 to interact with and disrupt lipid bilayers.

**Figure 1:**
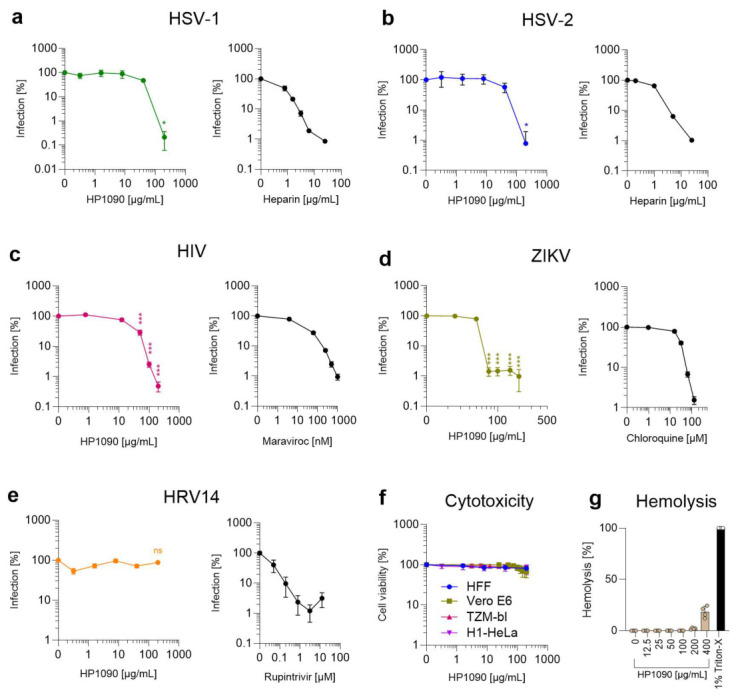
Antiviral activity of HP1090 and cytotoxicity of HP1090 on mammalian cell lines. Viruses were pre-incubated with the indicated concentrations of HP1090 or positive control compounds for 1 h at 37 °C, and the virus-compound mixtures were then added to target cells. (**a**) HSV-1 and (**b**) HSV-2 infection were tested on human foreskin fibroblasts (HFFs) with HP1090 or heparin. (**c**) HIV-1 infection was evaluated on TZM-bl reporter cells with HP1090 or maraviroc. (**d**) ZIKV infection was tested on Vero E6 cells with HP1090 or chloroquine. (**e**) HRV14 was assessed on H1-HeLa cells with HP1090 or rupintrivir. For each virus infection, the results with the HP1090 are shown on the left panel, and the control on the right panel. Infection levels are expressed as percentage of untreated control. (**f**) Cytotoxicity of HP1090 on HFF, Vero E6, TZM-bl, and H1-HeLa cells which were treated with increasing concentrations of HP1090 and cell viability was assessed by MTT assay. HFF and Vero E6 cells were treated for 2 days, while TZM-bl and H1-HeLa cells were treated for 3 days. (**g**) Hemolytic activity of HP1090 against human erythrocytes incubated with HP1090 (0–400 µg/mL) for 1 h at room temperature, normalized to 1% Triton X-100 (100% lysis). All peptide concentrations refer to on-virus concentration; approximately five-fold higher than on-cell concentration. Data represent mean ± SEM from three independent experiments each performed in triplicate. Statistical significance in panels (**a**–**d**) was determined by one-way ANOVA followed by Bonferroni’s multiple comparison test, comparing each treatment concentration to the untreated control. * *p* < 0.05, *** *p* < 0.001. For HRV14 (**e**), no significant (ns) dose-dependent effect was observed (test for linear trend, *p* = 0.27).

Given its previously reported antiviral activity against hepatitis C virus [8] and its predicted membrane-active properties, we sought to systematically evaluate its antiviral and antibacterial activity spectrum and to investigate the underlying mechanism of action. The antiviral activity of chemically synthesized HP1090 was assessed using cell-based infection assays against a panel of enveloped viruses, including herpes simplex virus type 1 (HSV-1), herpes simplex virus type 2 (HSV-2), human immunodeficiency virus type 1 (HIV-1), and Zika virus (ZIKV). Infection rates were quantified relative to untreated controls and normalized to the absence of peptide (Figure 1).

HP1090 inhibited HSV-1 infection in a dose-dependent manner, with a half-maximal inhibitory concentration (IC_50_) of 38.18 µg/mL (Figure 1a). HSV-2 infection was efficiently inhibited by HP1090 in a concentration-dependent fashion (Figure 1b), with an IC_50_ value of 40.68 µg/mL, comparable to that observed for HSV-1. The magnitude and shape of the dose–response curves were highly similar between the two herpesviruses. Heparin was included as a positive control to block viral attachment, consistently suppressing both HSV-1 and HSV-2 infections (Figure 1a,b), and confirmed assay performance. In HIV-1 infection assays, HP1090 displayed a clear dose-dependent antiviral activity, with an IC_50_ value of 14.71 µg/mL (Figure 1c), the lowest among the viruses tested. The CCR5 antagonist maraviroc was used as a reference control and fully suppressed infection under the same experimental conditions (Figure 1c). HP1090 also inhibited ZIKV infection in a concentration-dependent manner, with an IC_50_ value of 56.07 µg/mL (Figure 1d). Near-complete inhibition was achieved at 75 µg/mL, with no further reduction observed at higher peptide concentrations, indicating that maximal antiviral efficacy was reached below the cytotoxic threshold for Vero E6 cells. Chloroquine effectively suppressed ZIKV infection and served as a positive control (Figure 1d). To extend the analysis to respiratory enveloped viruses, HP1090 was additionally tested against hCoV-229E. Significant inhibition was observed at the highest concentration tested (200 µg/mL), indicating that HP1090 retains activity against this human coronavirus, albeit at lower potency than observed for HIV-1, HSV-1, HSV-2, and ZIKV (Supplementary Figure S5).

These data demonstrate that HP1090 is active against both DNA and RNA enveloped viruses and is not restricted to a single viral family. In contrast to its activity against enveloped viruses, HP1090 showed no antiviral activity against human rhinovirus 14 (HRV14) (Figure 1e), a non-enveloped virus. Across all tested concentrations, infection rates did not decrease, while the assay-specific positive control rupintrivir fully inhibited the infection (Figure 1e). These results demonstrate that the antiviral activity of HP1090 depends on the presence of a viral lipid envelope. Importantly, HP1090 was not cytotoxic on any of the tested cell lines at effective inhibitory concentrations (Figure 1f). In addition, HP1090 showed negligible hemolytic activity up to 200 µg/mL (Figure 1g) and modest hemolysis at 400 µg/mL, well above the maximum concentration used in the antiviral assays.

To further investigate the mechanism of antiviral activity, time-of-addition experiments were performed using ZIKV. HP1090 inhibited infection only when pre-incubated with virus particles prior to infection, whereas pre-treatment of cells followed by peptide removal or addition after viral adsorption resulted in little to no inhibition (Supplementary Figure S4). These findings support a direct virucidal mechanism and argue against major effects on host cells or post-entry stages of infection.

Collectively, these data establish HP1090 as a broad-spectrum antiviral peptide with selective activity against multiple enveloped viruses while exhibiting minimal activity against non-enveloped viruses and limited toxicity toward mammalian cells.

### 2.2. HP1090 Rapidly Disrupts Virus-like Lipid Membranes in a Liposome Leakage Assay

**Figure 2:**
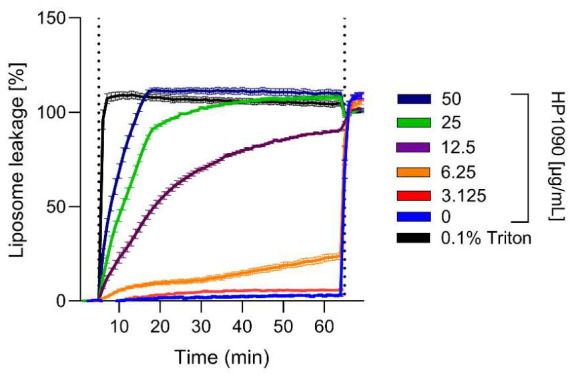
Liposome disruption triggered by HP1090 incubation. Carboxyfluorescein-loaded liposomes mimicking viral membrane composition (DOPC/sphingomyelin/cholesterol at 45:25:30 mol%) were incubated with the indicated concentrations of HP1090 as well as 0.1% of Triton X-100 as a positive control, and dye release was monitored by fluorescence over time. The first dotted line indicates the start of liposome incubation after the baseline was established by measuring fluorescence for 5 min and the second dotted line marks the addition of 1% Triton X-100 to all the samples after 60 min of incubation to measure maximum intensity. Data are expressed as percentage of maximum dye release (normalized to the leakage after the addition of Triton) and are shown as mean ± SEM derived from three independent experiments.

To directly assess whether HP1090 disrupts lipid bilayers resembling viral envelopes, we performed a liposome leakage assay using virus-like liposomes composed of phosphatidylcholine, sphingomyelin, and cholesterol. This lipid composition was chosen based on a previously established viral membrane model [10] and reflects the enrichment of sphingomyelin and cholesterol characteristic of lipid-raft-derived viral envelopes. Liposomes were loaded with carboxyfluorescein at self-quenching concentrations, enabling membrane disruption to be quantified as an increase in fluorescence upon dye release. Complete liposome lysis was induced by 1% Triton X-100 and defined as 100% leakage for normalization across experiments. Fluorescence was recorded at one-minute intervals over 60 min, allowing both the extent and kinetics of membrane disruption to be assessed (Figure 2). Addition of chemically synthesized HP1090 induced rapid and concentration-dependent release of carboxyfluorescein, indicating efficient disruption of liposomal membrane integrity. At higher peptide concentrations (25–50 µg/mL), fluorescence increased sharply within the first minutes after peptide addition and reached a plateau corresponding to substantial membrane leakage. Intermediate concentrations (12.5 µg/mL) resulted in slower but clearly detectable leakage over time, whereas lower concentrations (6.25 µg/mL) induced delayed and reduced fluorescence increases. The pronounced dose dependence and rapid kinetics of liposome leakage demonstrate that HP1090 directly compromises the integrity of virus-like lipid bilayers. Importantly, these effects were observed in the absence of cellular components, providing direct biophysical evidence that HP1090 acts through a membrane-disruptive mechanism.

### 2.3. HP1090 Inhibits Growth of Clinically Relevant and Multidrug-Resistant Bacterial Pathogens

**Figure 3:**
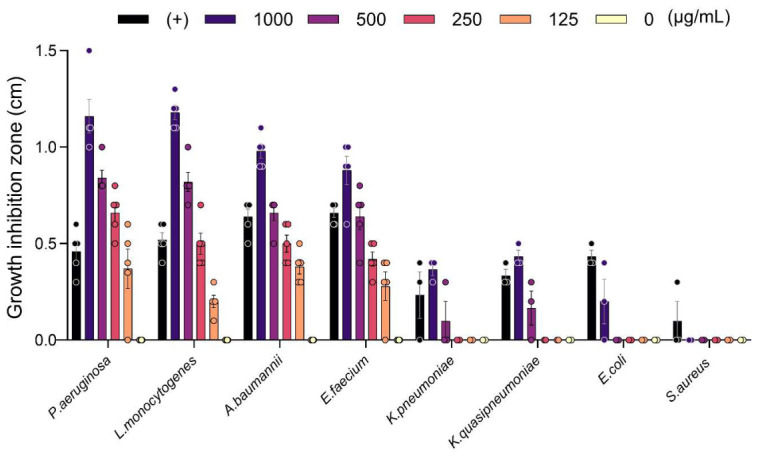
Antibacterial activity of HP1090. Growth inhibition of the indicated bacterial strains was assessed following treatment with HP1090 at concentrations ranging from 0 to 1000 μg/mL on agar plates. LL-37 (100 μg/mL) was used as a positive control (+). Growth inhibition zone measurements are expressed in cm. Data represent mean ± SEM from five independent experiments for *P. aeruginosa*, *L. monocytogenes*, *A. baumannii*, and *E. faecium* and three for all the rest.

Following the demonstration of membrane-disruptive activity in virus-like liposomes, the antibacterial activity of chemically synthesized HP1090 was evaluated against a panel of clinically relevant bacterial pathogens using agar-based growth inhibition assays (Figure 3). HP1090 was tested at concentrations ranging from 125 to 1000 µg/mL, which are comparable to the concentrations used in the antiviral and membrane-disruption experiments. The bacterial panel included selected ESKAPE pathogens that often cause severe infections with multi-drug-resistant strains [11], as well as *Listeria monocytogenes* an important pathogen in the food industry sector, that can be fatal in cases of pregnancy or immunocompromised individuals [12].

Within this concentration range, HP1090 produced clear growth inhibition zones against several bacterial species, including Gram-negative *Pseudomonas aeruginosa*, *Acinetobacter baumannii*, as well as Gram-positive vancomycin-resistant *Enterococcus faecium*, and *Listeria monocytogenes* (Figure 3). HP1090 demonstrated dose-dependent growth inhibition across most tested strains, including multidrug-resistant *Enterococcus* sp. (vancomycin-resistant), *K. pneumoniae*, and *K. quasipneumoniae* but showed no activity against methicillin-resistant *S. aureus* (MRSA). The extent of growth inhibition increased with peptide concentration and varied between species, indicating species-dependent susceptibility to HP1090. While antibacterial activity was not uniformly observed across all tested bacteria, these results demonstrate that HP1090 is active against multiple clinically relevant Gram-negative and Gram-positive pathogens at higher µg/mL concentrations. For comparison, the membrane-active antimicrobial peptide cathelicidin LL-37 [2] was included as a reference at 100 µg/mL. LL-37 displayed the expected antibacterial activity, serving as a positive control (Figure 3).

### 2.4. Antibacterial Activity of HP1090 Against L. monocytogenes at Lower pH

**Figure 4:**
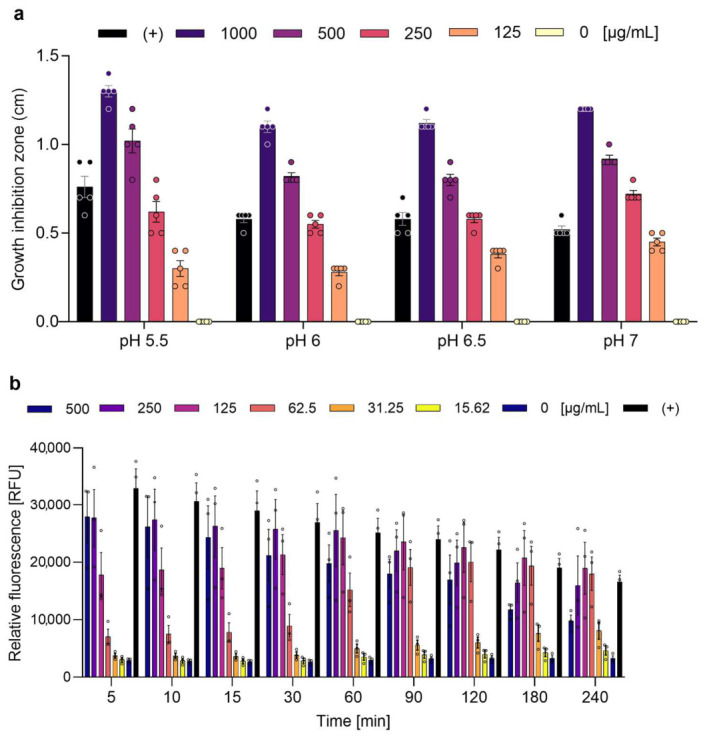
HP1090 exhibits antibacterial activity across different pH values and induces membrane permeabilization in *L. monocytogenes*. (**a**) Growth inhibition of *L. monocytogenes* was assessed at different pH values (5.5–7.0) following treatment with HP1090 at concentrations ranging from 0 to 1000 μg/mL. LL-37 (100 μg/mL) and H_2_O served as positive and negative controls, respectively. (**b**) Membrane permeabilization of *L. monocytogenes* was monitored using SYTOX Green fluorescence over time. Bacteria were treated with HP1090 at the indicated concentrations, or with ethanol and H_2_O as positive (+) and negative (0 µg/mL) controls, respectively. Data shown represent mean ± SEM from five independent experiments in (**a**) and three in (**b**). RFU, relative fluorescence units.

To assess whether environmental pH influences the antibacterial activity of chemically synthesized HP1090, growth inhibition assays against *L. monocytogenes* were performed at different pH values (Figure 4a). HP1090 was tested at concentrations ranging up to 1000 µg/mL, consistent with the concentrations used in the initial antibacterial screening. HP1090 inhibited *L. monocytogenes* growth across all tested pH conditions. Slightly larger inhibition zones were observed at acidic pH values. These results demonstrate that HP1090 retains antibacterial activity against *Listeria monocytogenes* across a physiologically relevant pH range and may exhibit some increased efficacy under acidic conditions, although pH values below 5.0 could not be evaluated, as *L. monocytogenes* did not grow reliably under these conditions in the radial diffusion format.

### 2.5. HP1090 Induces Rapid Membrane Permeabilization of L. monocytogenes

To directly assess whether the antibacterial activity of chemically synthesized HP1090 is associated with disruption of bacterial membrane integrity, membrane permeabilization of *Listeria monocytogenes* was analyzed using a SYTOX Green uptake assay (Figure 4b). SYTOX Green is excluded from intact cells but penetrates cells with compromised membranes, resulting in increased fluorescence upon binding to intracellular nucleic acids. Fluorescence was monitored over time and compared to a positive control inducing complete membrane permeabilization and a negative control representing intact cells.

Treatment of *L. monocytogenes* with HP1090 resulted in an increase in SYTOX fluorescence, indicating membrane permeabilization. These findings provide direct experimental evidence that HP1090 compromises the bacterial membrane of *Listeria monocytogenes*. Together with the growth inhibition and pH-dependence data, the SYTOX assay supports a membrane-disruptive mode of action underlying the antibacterial activity of HP1090.

## 3. Discussion

In this study, we provide a systematic functional characterization of the scorpion venom peptide HP1090 and demonstrate that its antiviral and antibacterial activities are consistent with a membrane-targeting mode of action. Venom-derived peptides are well recognized as a rich source of bioactive molecules with diverse biological functions, including antimicrobial and cytolytic activities [5,6]. Within this context, HP1090 represents a member of the non-disulfide-bridged, amphipathic α-helical peptides that are thought to contribute to innate defense mechanisms in scorpions and other arthropods [5,8].

While HP1090 was originally described as a virucidal peptide active against hepatitis C virus [8], its broader antiviral spectrum and antibacterial activity have not been systematically investigated. Here, we show that HP1090 inhibits infection by multiple phylogenetically distinct enveloped viruses, including HSV-1, HSV-2, HIV-1, and Zika virus. The absence of activity against the non-enveloped virus HRV14 provides strong functional evidence that the viral lipid envelope is required for activity. As all antiviral experiments were performed under pre-incubation conditions, these results indicate that HP1090 acts directly on viral particles prior to host cell entry, consistent with a virucidal mode of action. Similar envelope-dependent effects have been described for other membrane-active peptides derived from venom or innate immunity [3,13,14].

This envelope dependence aligns with the general behavior of amphipathic venom-derived peptides, which frequently exert their biological activity through direct interaction with lipid membranes [1,2]. In contrast to receptor-targeting or enzyme-inhibiting toxins, such peptides act via physicochemical interactions that depend on membrane composition, curvature, and lipid packing [4,15]. The broad antiviral activity observed across unrelated viruses in this study supports a mechanism that relies on conserved biophysical properties of viral membranes rather than virus-specific entry pathways.

A key aspect of this work is the integration of cellular infection assays with cell-free membrane models. Using virus-like liposomes, we demonstrate that HP1090 induces rapid and concentration-dependent membrane permeabilization, providing direct experimental support for membrane disruption as a primary mode of action. However, while the leakage kinetics are consistent with membrane destabilization, the precise molecular mechanism, such as pore formation, carpet-like disruption, or detergent-like solubilization, cannot be distinguished based on the current data [15,16,17]. Such mechanistic resolution would require high-resolution structural or biophysical approaches and remains an important objective for future studies.

The concentrations that induced liposome leakage were in the same general range as those required for antiviral activity, supporting membrane permeabilization as a major contributor to viral inhibition. However, the relationship between membrane leakage and biological activity is not strictly quantitative. The antiviral IC_50_ values differed among viruses, and antibacterial activity varied substantially among bacterial species despite the common membrane-targeting mechanism proposed here. These differences likely reflect variations in membrane composition, curvature, packing stress, protein content, cell envelope architecture, and peptide-to-membrane ratios. Thus, HP1090 does not appear to target a specific viral family or bacterial species. Rather, susceptibility is likely determined by the biophysical properties of the target membrane, which vary across experimental systems and biological targets.

The ability of venom-derived peptides to discriminate between target and host membranes is a critical determinant of their biological function. HP1090 exhibited measurable selectivity, with limited cytotoxic effects on mammalian cells at concentrations effective for antiviral activity. While differences in membrane composition, including lipid charge, cholesterol content, and membrane organization, likely contribute to this selectivity, lipid composition alone is unlikely to fully explain the observed preference for viral membranes [15,18]. Indeed, the DOPC/sphingomyelin/cholesterol liposomes employed in this study represent a widely used model system for membrane permeabilization assays and approximate the sphingolipid- and cholesterol-rich composition of many viral envelopes [10,19]. However, viral envelopes share many lipid constituents with mammalian plasma membranes. Additional factors may therefore contribute to the selective antiviral activity of HP1090. For example, the high membrane curvature and associated packing stress of viral particles may increase their susceptibility to peptide-mediated membrane perturbation compared with the relatively flat membranes of mammalian cells. Differences in peptide-to-membrane ratios may further result in higher local peptide densities on viral particles and liposomes than on the much larger surface area of intact cells. Moreover, unlike viral particles, living cells possess active membrane repair mechanisms that can counteract transient membrane damage. Viral envelopes and bacterial membranes typically lack the asymmetric lipid distribution and repair mechanisms characteristic of mammalian cells, rendering them more susceptible to membrane-active peptides [2,20]. At the same time, the limited margin between effective antiviral and cytotoxic concentrations indicates that HP1090, like many naturally occurring venom peptides, is not fully optimized for therapeutic selectivity.

In addition to its antiviral effects, HP1090 displayed antibacterial activity against several clinically relevant pathogens, including vancomycin-resistant *E. faecium*, a multidrug-resistant (MDR) strain. However, antibacterial activity required substantially higher concentrations and was not uniformly observed across all tested species, with no detectable activity against MRSA. These differences likely reflect variations in bacterial envelope architecture, including the presence of outer membranes in Gram-negative bacteria and differences in cell wall composition in Gram-positive organisms [4,20]. The antibacterial activity demonstrated here is supported by membrane permeabilization of *L. monocytogenes*, indicating that disruption of bacterial membrane integrity contributes to the observed growth inhibition. Nevertheless, the radial diffusion assay used here provides primarily qualitative, screening-level evidence of antibacterial activity, and the results should be interpreted accordingly. Quantitative determination of minimum inhibitory concentrations (MICs) will be required to fully define the antibacterial potency and spectrum of HP1090 and to enable direct comparison with established antimicrobial peptides [21].

From a toxin biology perspective, the dual antiviral and antibacterial activity of HP1090 highlights the functional versatility of short amphipathic venom peptides. Such peptides are thought to play a role in protecting venom-producing organisms from microbial infection and may contribute to prey immobilization or defense [6]. The relatively low net charge and short length of HP1090 distinguish it from many classical antimicrobial peptides and may influence both its activity spectrum and selectivity profile [5,9].

Compared to other well-characterized membrane-active peptides, HP1090 is notably short (13 amino acids) and carries a relatively low net positive charge (+2), yet retains dual antiviral and antibacterial activity. By contrast, the human cathelicidin LL-37, used here as a reference compound, comprises 37 residues with a net charge of +6 and requires its full length for optimal antimicrobial function [2]. Similarly, melittin, a 26-residue bee venom peptide with a net charge of +6, exhibits potent membrane-lytic activity but is associated with substantial hemolytic toxicity that limits its therapeutic utility [22]. The scorpion-derived mucroporin-M1, a closer structural analogue at 17 residues, has demonstrated antiviral activity against measles virus, SARS-CoV, and influenza H5N1 [14], but its antibacterial spectrum has not been systematically evaluated alongside its antiviral properties. HP1090 thus combines a minimal peptide scaffold with demonstrable activity against both enveloped viruses and clinically relevant bacteria, offering potential advantages in terms of synthetic accessibility and cost of production. However, its moderate potency relative to longer, more highly charged peptides underscores the need for sequence optimization to improve activity while preserving its favorable selectivity profile.

From a translational standpoint, the effective antiviral concentrations of HP1090 fall within the range achievable in topical antimicrobial formulations, suggesting that local application may represent a potential future use case. However, the present study did not address several parameters critical for therapeutic development, including peptide stability, protease resistance, serum activity, pharmacokinetics, or in vivo efficacy. Although HP1090 exhibited limited cytotoxicity and no substantial hemolytic activity at antiviral concentrations, further studies will be required to determine whether these activities are maintained under physiologically relevant conditions. Consequently, therapeutic applicability should currently be regarded as a future possibility rather than a direct implication of the present findings. For broader development, optimization strategies such as sequence modification, stabilization, or targeted delivery may further enhance potency and selectivity [2,20].

## 4. Conclusions

In summary, our findings establish HP1090 as a membrane-active scorpion venom peptide with broad antiviral activity against enveloped viruses. The antiviral effects correlate with membrane disruption in model membrane systems, supporting a membrane-targeted mode of action. In addition, HP1090 exhibited antibacterial activity in radial diffusion assays and induced membrane permeabilization in bacterial cells, suggesting that membrane interactions may also contribute to its effects on bacteria. However, further studies employing quantitative antibacterial assays will be required to define its antibacterial potency and spectrum more rigorously.

## 5. Materials and Methods

### 5.1. Viral Experiments

#### 5.1.1. Cell Culture

Vero E6 cells (ATCC) under passage 20 were cultured in DMEM supplemented with 2.5% (*v*/*v*) heat-inactivated FCS, 2 mM L-glutamine, 1 mM sodium pyruvate, 1× non-essential amino acids, 100 units/mL penicillin, and 100 µg/mL streptomycin. H1-HeLa cells (ATCC) under passage 10 were cultured in DMEM supplemented with 10% heat-inactivated FCS, 2 mM L-glutamine, 100 units/mL penicillin, and 100 µg/mL streptomycin. Human foreskin fibroblasts (HFFs) were provided by Jens von Einem (Institute of Virology, Ulm University Medical Center, Ulm, Germany) and cultured in DMEM supplemented with 10% heat-inactivated FCS, 2 mM L-glutamine, 100 units/mL penicillin, and 100 µg/mL streptomycin and were used under passage 10. TZM-bl cells are CD4+, CCR5+, and CXCR4+ HeLa-derived HIV-1 reporter cells encoding firefly luciferase and lacZ genes under control of the HIV-1 LTR promoter. TZM-bl cells were obtained from the National Institutes of Health AIDS Research and Reference Reagent Program (ARRRP) (now part of BEI resources, National Institute of Health, Manassas, VA, USA) and cultured in DMEM supplemented with 10% heat-inactivated FCS, 2 mM L-glutamine, 100 units/mL penicillin, and 100 µg/mL streptomycin and experiments were performed under passage 10. Huh-7 cells, a hepatocyte-derived carcinoma cell line provided by Anna-Laura Kretz (Department of General and Visceral Surgery, Ulm University), were used under passage 20 and maintained in DMEM supplemented with 100 U/mL penicillin, 100 μg/mL streptomycin, 2 mM L-glutamine, and 10% heat-inactivated FCS. All cell lines were maintained at 37 °C and 5% CO_2_ in a T175 flask, split when reaching 80–90% confluency and regularly tested for mycoplasma.

#### 5.1.2. Cell Viability Assay

Cytotoxicity of HP1090 was assessed using an MTT-based cell viability assay. MTT (3-(4,5-dimethylthiazol-2-yl)-2,5-diphenyltetrazolium bromide) assays were performed in parallel to the antiviral assays, with cell culture medium added in place of virus. Medium was removed and 100 µL of MTT stock (5 mg/mL in PBS, diluted 1:10) was added per well. After 2.5 h of incubation at 37 °C, the supernatant was discarded and formazan crystals were dissolved in 100 µL of 1:1 DMSO:ethanol. Absorbance was measured at 450 nm with baseline correction at 650 nm using a Vmax microplate reader (Molecular Devices, San Jose, CA, USA). Untreated controls were set to 100% viability.

#### 5.1.3. Hemolysis ASSAY

Hemolytic activity of HP1090 was assessed using human erythrocytes from healthy donors. Whole blood (5 mL) was centrifuged at 1000× *g* for 10 min at 4 °C, and the serum was removed. Erythrocytes were resuspended and diluted 1:20 in PBS. HP1090 was serially diluted 1:2 in PBS in a V-bottom 96-well plate, starting from a maximum concentration of 400 µg/mL. Diluted erythrocytes were added to each well and plates were incubated for 1 h at room temperature with shaking at 250 rpm. After incubation, plates were centrifuged at 1500 rpm for 5 min, and 35 µL of supernatant was transferred to a flat-bottom 96-well plate. Hemoglobin release was measured by absorbance at 405 nm using a plate reader (Molecular Devices, San Jose, CA, USA). PBS-treated erythrocytes served as the negative control (0% hemolysis) and 1% Triton X-100 as the positive control (100% hemolysis). Absorbance values were blank-corrected against the PBS control and normalized to the Triton X-100 condition. Data represent mean ± SEM from four independent donors, each performed in triplicate.

#### 5.1.4. Peptide Synthesis

Synthetic HP1090 was produced by solid-phase Fmoc chemistry using a Liberty Blue microwave peptide synthesizer (CEM Corporation, Matthews, NC, USA). Peptide identity and purity was confirmed by reversed-phase chromatographic analysis (Supplementary Figure S2) and MALDI-TOF mass spectrum (Supplementary Figure S3).

The reversed-phase chromatographic analysis was performed on a Biobasic C18 RP-HPLC column (Thermo Scientific, Waltham, MA, USA) of dimensions 2.1 × 100 mm (5 µm) at 0.5 mL/min using the gradient 0/5, 10/45, and 12/100 (total running time in min/%B), with A being 0.1% TFA/water, and B, 0.1% TFA/acetonitrile. All separations were run on an Agilent 1100 HPLC system (Agilent Technologies, Santa Clara, CA, USA). Online UV detection was performed at 225 nm. Chromatogram recording and peak integration were done with ChemStation B.04.03 (Agilent Technologies, Santa Clara, CA USA).

Synthetic HP1090 was analyzed with an Axima Confidence MALDI-TOF MS (Shimadzu, Kyoto, Japan) in positive reflector mode on a 384-spot stainless-steel sample plate. Spots were coated with 1 µL of 10 mg/mL CHCA dissolved in a mixture of TFA/water/2-propanol/acetonitrile (2.5/47.5/25/25), and the solvent was allowed to air-dry. Subsequently, 0.5 µL of sample or standard was applied to the dry, pre-coated well and immediately mixed with 0.5 µL of matrix; the solvent was allowed to air dry. All spectra were acquired in the positive ion linear mode using a 337-nm N_2_ laser. Ions were accelerated from the source at 20 kV. A hundred profiles were acquired per sample, and 20 shots were accumulated per profile. The equipment was calibrated with a standard mixture provided in the TOFMix^TM^ MALDI kit (Shimadzu, Kyoto, Japan). Measurements and MS data processing were controlled by the MALDI-MS Application Shimadzu Biotech Launchpad 2.9.8.1 (Shimadzu, Kyoto, Japan).

#### 5.1.5. Effect of HP1090 on HSV-1 and HSV-2 Infection

HSV-1-eGFP (F strain) was provided by Benedikt Kaufer (Free University of Berlin, Berlin, Germany). The eGFP expression cassette is inserted between the convergently transcribed UL3 and UL4 genes. HSV-2-eGFP (strain 333) was provided by Patricia Spear (Northwestern University, Chicago, IL, USA) and was generated by co-transfecting Vero E6 cells with genomic DNA from wildtype HSV-2 (333) and plasmid pUL3UL4-CMVeGFP. Virus stocks of HSV-1 and HSV-2 were generated by infecting Vero E6 cells. After 3–5 days, cell supernatants were centrifuged at 330× *g* for 3 min to remove cell debris, and virus stocks were stored at −80 °C.

For infection assays, 6000 HFFs were seeded into 96-well plates one day prior to infection. HSV-1-eGFP and HSV-2-eGFP at a MOI of 0.2 were pre-incubated with HP1090 (0–200 µg/mL) or heparin (0–25 µg/mL) for 1 h at 37 °C in triplicate prior to addition to HFFs. Infection rates were determined two days post-infection by quantifying GFP-positive cells using a CytoFLEX flow cytometer and CytExpert 2.3 software (Beckman Coulter, Brea, CA, USA). Uninfected cells were used to establish the gating threshold for the GFP signal, and the percentage of GFP-positive cells served as a quantitative readout for relative infection.

#### 5.1.6. Effect of HP1090 on Zika Virus Infection

Virus stocks of ZIKV MR766, a strain originally isolated from a sentinel rhesus macaque in 1947 [23], were provided by Jonas Schmidt-Chanasit (Bernhard Nocht Institute for Tropical Medicine, Hamburg, Germany) and generated by infecting Vero E6 cells. After 3–5 days, cell supernatants were centrifuged at 330× *g* for 3 min to remove cell debris, and virus stocks were stored at −80 °C.

For infection assays, 6000 Vero E6 cells were seeded into 96-well plates one day prior to infection. ZIKV MR766 at a MOI of 0.5 was pre-incubated with HP1090 (0–200 µg/mL) or chloroquine (0–125 µM) for 1 h at 37 °C in triplicate prior to addition to Vero E6 cells. Two days post-infection, infection rates were determined using a cell-based ZIKV immunodetection assay [19]. Cells were washed with PBS and fixed with 4% paraformaldehyde (PFA) for 20 min at room temperature. Permeabilization was performed with cold methanol for 5 min at 4 °C, followed by washing with PBS. Cells were then incubated with mouse anti-flavivirus antibody 4G2 (1:10,000; Absolute Antibodies, Newark, CA, USA, AB-00230-2.0) in antibody buffer (PBS containing 10% FCS and 0.3% Tween-20) for 1 h at 37 °C, washed three times with washing buffer (PBS containing 0.3% Tween-20), and incubated with an HRP-conjugated anti-mouse secondary antibody (1:20,000; Thermo Fisher Scientific, Waltham, MA, USA, A16066) for 1 h at 37 °C. After four washes, TMB peroxidase substrate was added. After 5 min at room temperature, the reaction was stopped with 0.5 M sulfuric acid, and absorbance was measured at 450 nm with baseline correction at 650 nm using a Vmax microplate reader (Molecular Devices, San Jose, CA, USA). Values were corrected for background signal from uninfected cells and are expressed as mean ± SEM from three independent experiments, each performed in triplicate.

#### 5.1.7. Effect of HP1090 on HIV-1 Infection

Virus stocks of CCR5-tropic HIV-1 NL4-3 92TH014.12 [24] were obtained from the NIH AIDS Reagent Program. To generate virus stocks, 9 × 10^5^ HEK293T cells were seeded into 6-well tissue culture plates (Sarstedt, Nümbrecht, Germany) 24 h before transfection. Cells were transfected with 2.5 µg of plasmid DNA per well using TransIT-LT1 (Mirus Bio, Madison, WI, USA) according to the manufacturer’s instructions. Virus stocks were harvested 48 h post-transfection and stored at −80 °C.

For infection assays, the reporter cell line TZM-bl was used. In the TZM-bl system, HIV-1 infection drives Tat-dependent activation of the LTR, resulting in β-galactosidase expression. 10,000 TZM-bl cells were seeded into 96-well plates one day prior to infection. HIV-1 was pre-incubated with HP1090 (0–200 µg/mL) or maraviroc (0–1000 nM) for 1 h at 37 °C in triplicate prior to addition to TZM-bl cells. Infection rates were determined three days post-infection by measuring β-galactosidase activity in cell lysates using the Gal-Screen β-Galactosidase Reporter Gene Assay System (Thermo Fisher Scientific, Waltham, MA, USA) and an Orion II microplate luminometer (Berthold, Bad Wildbad, Germany). Values represent reporter gene activity (RLU/s) from triplicate, corrected for background from uninfected cells, and are expressed as mean ± SEM from three independent experiments.

#### 5.1.8. Effect of HP1090 on Human Rhinovirus 14

Virus stocks of human rhinovirus 14 (HRV14; ATCC, #VR-284) were propagated by infecting H1-HeLa cells at 33 °C in medium containing 2% FCS. After three days, cell supernatants were centrifuged at 330× *g* for 5 min to remove cell debris, and virus aliquots were stored at −80 °C.

For infection assays, 6000 H1-HeLa cells were seeded into 96-well plates one day prior to infection in medium containing 2% FCS. HRV14 at a MOI of 0.1 was pre-incubated with HP1090 (0–200 µg/mL) or rupintrivir (0–12.5 µM) for 1 h at 37 °C in triplicate prior to addition to H1-HeLa cells. Infection rates were determined three days post-infection using the CellTiter-Glo Luminescent Cell Viability Assay (Promega, Madison, WI, USA) and an Orion II microplate luminometer (Berthold, Bad Wildbad, Germany). Values represent luminescence (RLU/s) from triplicate, corrected for background from uninfected cells.

#### 5.1.9. Effect of HP1090 on hCoV229E

HCoV-229E (ATCC, VR-740) was propagated on HuH7 cells inoculated at a MOI of 0.1 in DMEM supplemented with 2% FCS at 33 °C. Cells were washed with PBS one day post-infection and cultured in fresh medium until strong cytopathic effects were visible (day 5). Supernatants were harvested, aliquoted, and stored at −80 °C.

For infection assays, 25,000 HuH7 cells were seeded into 96-well plates one day prior to infection. HCoV-229E at a MOI of 0.02 was pre-incubated with HP1090 (0–200 µg/mL) or remdesivir (0–10 µM) for 1 h at 33 °C in triplicate prior to addition to HuH7 cells. Two days post-infection, infection rates were determined by cell-based immunodetection as described for ZIKV, using anti-229E (40640-T62, Sino Biological, Beijing, China) at a dilution of 1:7500 as the primary antibody and HRP-coupled anti-rabbit antibody (#31460, Thermo Fisher, Waltham, MA, USA) as the secondary antibody.

#### 5.1.10. Statistical Analysis

Statistical analysis was performed using GraphPad Prism (version 10.6.1; GraphPad Software, San Diego, CA, USA). For antiviral and cytotoxicity assays, differences between treatment groups were assessed by one-way ANOVA followed by Bonferroni’s multiple comparison test. For HRV14, where no dose-dependent antiviral effect was expected, a test for linear trend was applied to evaluate whether infection rates decreased systematically with increasing peptide concentration. Data are presented as mean ± SEM from three independent experiments, each performed in triplicate. Differences were considered statistically significant at *p* < 0.05.

#### 5.1.11. Liposome Leakage Assay

The liposome leakage assay was performed as previously described [10]. Liposomes were prepared by thin-film hydration and extrusion. Lipids (Avanti Polar Lipids, Alabaster, AL, USA) consisting of DOPC, egg sphingomyelin, and ovine cholesterol (45:25:30 mol%) were hydrated by adding 1 mL of 50 mM 5(6)-carboxyfluorescein prepared in 50% PBS (iso-osmolar to PBS), adjusted to pH 7.4 with NaOH, yielding an approximate total lipid concentration of 5 mM. The glass vials were shaken at 60 °C and 180 rpm for 3 h. Small unilamellar vesicles were prepared by 25 extrusions through 0.2 µm polycarbonate membranes (Nuclepore Track-Etched Membrane; Whatman, Maidstone, UK) using a Mini Extruder (Avanti Polar Lipids) on a heating platform at 70 °C. Free dye was removed by two sequential size-exclusion filtration steps using PD MidiTrap Sephadex G-25 columns (GE Healthcare, Chicago, IL, USA), and liposomes were quantified by nanoparticle tracking analysis (NTA) using a ZetaView instrument (Particle Metrix, Inning am Ammersee, Germany). Liposome preparations were diluted in PBS, and 2.25 × 10^9^ particles per well were added in a volume of 90 µL.

Fluorescence intensity was measured at an excitation wavelength of 493 nm and emission wavelength of 517 nm using a Synergy H1 plate reader and Gen5 software (BioTek Instruments, Winooski, VT, USA). The baseline was established by measuring fluorescence for 5 min, after which 10 µL of compound was added and plates were incubated at 37 °C for 1 h with measurements recorded every minute. Maximum dye release (100%) was determined by adding Triton X-100 to a final concentration of 1%, followed by an additional 5 min of measurement.

#### 5.1.12. Time-of-Addition Assay of HP1090

To determine the stage at which HP1090 exerts its antiviral activity, time-of-addition experiments were performed using ZIKV and Vero E6 cells seeded at 6000 cells per well in a 96-well plate. Three experimental configurations were tested: (i) Pre-incubation: HP1090 (0–200 µg/mL) was incubated with ZIKV at a MOI of 0.5 for 1 h at 37 °C prior to addition to Vero E6 cells; (ii) Virus first: Vero E6 cells were infected with ZIKV for 1 h at 37 °C, after which HP1090 (0–200 µg/mL) was added directly to the infected cells; (iii) Peptide first: Vero E6 cells were pre-treated with HP1090 (0–200 µg/mL) for 1 h at 37 °C, washed with PBS three times to remove peptide, and subsequently infected with ZIKV. In all conditions, infection rates were determined two days post-infection by cell-based ZIKV immunodetection as described above. All concentrations refer to the on-virus pre-incubation scale; on-cell concentrations are approximately five-fold lower. Data represent a single experiment performed in triplicate.

### 5.2. Bacterial Experiments

#### 5.2.1. Bacterial Culture

All bacterial strains used in this study are listed in Table 1. For liquid culture, *Escherichia coli*, *Pseudomonas aeruginosa*, and *Acinetobacter baumannii* were grown in 10 mL lysogeny broth (LB-Miller) at 37 °C with shaking (160 rpm) under aerobic conditions. Methicillin-Resistant *Staphylococcus aureus*, *Klebsiella pneumoniae*, *Klebsiella quasipneumoniae*, *Enterococcus faecium* (VRE) and *Listeria monocytogenes* were cultured in 10 mL THY medium (Todd-Hewitt Broth (Oxoid) supplemented with 0.5% yeast extract (BD, Miami, FL, USA) at 37 °C in a 5% CO_2_ atmosphere).

#### 5.2.2. Radial Diffusion Assays

To screen and evaluate the antibacterial activity of HP1090 against the ESKAPE pathogens, a radial diffusion assay (RDA) was carried out. An overnight bacterial culture was pelleted by centrifugation at 3000× *g* and washed in 10 mM sodium phosphate buffer. Following another centrifugation, the pellet was resuspended in 10 mM sodium phosphate buffer, and the optical density was determined spectrophotometrically at O.D. 600 nm. Bacterial density was adjusted to seed approximately 2 × 10^7^ bacteria into 15 mL of 1% agarose. Plates were poured and allowed to cool for 30 min at 4 °C, then 2–3 mm holes were placed in the agarose using sterile wide-pore pipette tips (Axygene, a Corning brand, Tewksbury, MA, USA). 10 µL of the peptide of the desired concentration was applied into the holes. Plates were incubated at 37 °C in ambient air for 3 h, then overlaid with 10 mL tryptic soy agar. After overnight incubation at 37 °C in 5% CO_2_, the diameters of clearly visible inhibition zones around the holes were determined manually using a measuring stick. The evaluation of positive activity of *P. aeruginosa*, *A. baumannii*, VRE, and *L. monocytogenes* was conducted in five independent experiments, while screening the antibacterial activity of *E. coli*, MRSA, *K. pneumoniae*, and *K. quasipneumoniae* was performed in three independent experiments.

To determine the effect of pH on the antimicrobial efficacy of HP1090, a pH RDA was performed against *L. monocytogenes*. The agarose and overlay tryptic soy agars were adjusted to various pH levels (5.5, 6, 6.5, and 7) using NaOH and HCl. The preparation of the bacteria and plates is described in detail in the RDA section above. After overnight incubation at 37 °C in 5% CO_2_, the diameters of clearly visible inhibition zones around the holes were measured manually using a measuring stick. Five independent experiments were conducted. Lower pH values (≤5.0) were not tested due to unreliable bacterial growth under these conditions in the radial diffusion assay format.

#### 5.2.3. Sytox Assays

To assess the antibacterial activity of HP1090 on the membrane integrity of *L. monocytogenes*, a SYTOX Green membrane permeabilization assay was performed. An overnight culture of *L. monocytogenes* was inoculated into fresh medium and grown to an optical density (OD) of 0.1 at 600 nm. Bacterial cells were harvested by centrifugation at 10,000× *g* for 2 min, and the pellets were resuspended in 0.9% NaCl containing 0.4 µM SYTOX Green dye (Invitrogen, Waltham, MA, USA). Bacterial cells treated with 70% ethanol served as a positive control for membrane permeabilization, while untreated cells served as a negative control.

A total of 50 µL of 0.4 µM SYTOX-NaCl suspension was transferred into each well of a black 96-well plate, except for the blank well and the first well. 100 µL and 80 µL of a 0.4 µM SYTOX-NaCl suspension were loaded into the blank well and the first well, respectively. To perform a serial dilution (1:2), 20 µL of 5 mg/mL of HP1090 peptide was added to the first well, then 50 µL was transferred and resuspended into the next wells to reach a decreasing concentration of the peptide (500–15.625 µg/mL). To reach an end volume of 100 µL, 50 µL of SYTOX-stained bacteria was added to each well. Fluorescence was measured immediately for four hours using a Tecan Infinite M plate reader with an excitation wavelength of 488 nm and corresponding emission settings. All conditions were tested in duplicate across three independent experiments.

## Figures and Tables

**Table 1:** List of bacterial strains used and their sources.

Bacterial Strain	Source
*Pseudomonas aeruginosa* (BSU 856)	ATCC 27853
*Escherichia coli* (BSU 1286)	Clinical isolate (gastrointestinal tract)
Methicillin-Resistant *Staphylococcus aureus* (BSU 1348)	ATCC 43300
*Klebsiella quasipneumoniae* (BSU 1353)	ATCC 700603
*Klebsiella pneumoniae* (BSU 2231)	DSM 30104
*Acinetobacter baumannii* (BSU 1514)	ATCC 19606
*Enterococcus faecium* VRE (BSU 1516)	DSM 17050
*Listeria monocytogenes* (BSU 1423)	Provided by C. Riedl (Ulm University)

## Data Availability

The original contributions presented in this study are included in the article. Further inquiries can be directed to the corresponding author.

## Supplementary Materials

The following supporting information can be downloaded at: https://www.mdpi.com/article/10.3390/toxins18060268/s1, Supplementary Figure S1. Sequence and structural properties of HP1090. (a) Physicochemical properties of HP1090. (b) Helical wheel projection showing amphipathic distribution of hydrophobic (yellow), polar/uncharged (green), and polar/basic (red) residues. Supplementary Figure S2. Reversed-phase chromatographic analysis of synthetic HP1090. Red trace: blank run. Blue trace: HP1090 run. Peak integration shows a purity of 96.348 %. Supplementary Figure S3. MALDI-TOF mass spectrum of synthetic HP1090. The m/z value (1509.706) closely matches the theoretical one (1509.88). Supplementary Figure S4. Time-of-addition assay of HP1090 on ZIKV-infected Vero E6 cells. Three experimental configurations were used to assess the stage of HP1090 antiviral activity. Pre-incubation: HP1090 was incubated directly with ZIKV for 1 h at 37 °C prior to addition to Vero E6 cells. On cell—virus first: Vero E6 cells were infected with ZIKV for 1 h, followed by addition of HP1090. On cell—peptide first: HP1090 was added to Vero E6 cells for 1 h, cells were washed with PBS to remove peptide, and subsequently infected with ZIKV. All concentrations refer to on virus concentration (on-cell concentration is five-fold lower, similar to the pre-incubation experiment). Infection was quantified by a cell-based ZIKV immunodetection assay two days post-infection and normalized to untreated controls. Data represent mean ± SEM from a single experiment performed in triplicate. Supplementary Figure S5. Antiviral activity of HP1090 against human coronavirus 229E (HCoV-229E) and cytotoxicity on HuH7 cells. (a) HCoV-229E was pre-incubated with the indicated concentrations of HP1090 (left) or remdesivir (right) for 1 h at 33 °C prior to addition to HuH7 cells. Infection was quantified by cell-based immunodetection and normalized to untreated controls. IC_50_ >100 µg/mL. (b) Cytotoxicity of HP1090 on HuH7 cells assessed by MTT assay. Data represent mean ± SEM from three independent experiments, each performed in triplicate.

## References

[1] Hancock R.E.W., Sahl H.G. (2006). Antimicrobial and Host-Defense Peptides as New Anti-Infective Therapeutic Strategies. Nat. Biotechnol..

[2] Mookherjee N., Anderson M.A., Haagsman H.P., Davidson D.J. (2020). Antimicrobial Host Defence Peptides: Functions and Clinical Potential. Nat. Rev. Drug Discov..

[3] Jenssen H., Hamill P., Hancock R.E.W. (2006). Peptide Antimicrobial Agents. Clin. Microbiol. Rev..

[4] Bahar A.A., Ren D. (2013). Antimicrobial Peptides. Pharmaceuticals.

[5] Almaaytah A., Albalas Q. (2014). Scorpion Venom Peptides with No Disulfide Bridges: A Review. Peptides.

[6] Harrison P.L., Abdel-Rahman M.A., Miller K., Strong P.N. (2014). Antimicrobial Peptides from Scorpion Venoms. Toxicon.

[7] Xia Z., He D., Wu Y., Kwok H.F., Cao Z. (2023). Scorpion Venom Peptides: Molecular Diversity, Structural Characteristics, and Therapeutic Use from Channelopathies to Viral Infections and Cancers. Pharmacol. Res..

[8] Yan R., Zhao Z., He Y., Wu L., Cai D., Hong W., Wu Y., Cao Z., Zheng C., Li W. (2011). A New Natural α-Helical Peptide from the Venom of the Scorpion *Heterometrus petersii* Kills HCV. Peptides.

[9] Tonk M., Valdés J.J., Cabezas-Cruz A., Vilcinskas A. (2021). Potent Activity of Hybrid Arthropod Antimicrobial Peptides Linked by Glycine Spacers. Int. J. Mol. Sci..

[10] Weil T., Groß R., Röcker A., Bravo-Rodriguez K., Heid C., Sowislok A., Le M.-H., Erwin N., Dwivedi M., Bart S.M. (2020). Supramolecular Mechanism of Viral Envelope Disruption by Molecular Tweezers. J. Am. Chem. Soc..

[11] Rice L.B. (2008). Federal Funding for the Study of Antimicrobial Resistance in Nosocomial Pathogens: No ESKAPE. J. Infect. Dis..

[12] Radoshevich L., Cossart P. (2018). Listeria monocytogenes: Towards a Complete Picture of Its Physiology and Pathogenesis. Nat. Rev. Microbiol..

[13] Gao B., Zhu S. (2010). Mucroporin-M1 Inhibits HIV-1 Infection by Blocking Viral Fusion and Entry. FEBS Lett..

[14] Li Q., Zhao Z., Zhou D., Chen Y., Hong W., Cao L., Yang J., Zhang Y., Shi W., Cao Z. (2011). Virucidal Activity of a Scorpion Venom Peptide Variant Mucroporin-M1 against Measles, SARS-CoV and Influenza H5N1 Viruses. Peptides.

[15] Matsuzaki K. (1999). Why and How Are Peptide–Lipid Interactions Utilized for Self-Defense? Magainins and Tachyplesins as Archetypes. Biochim. Biophys. Acta.

[16] Lee T.H., Hall K.N., Aguilar M.I. (2016). Antimicrobial Peptide Structure and Mechanism of Action: A Focus on the Role of Membrane Structure. Curr. Top. Med. Chem..

[17] Brogden K.A. (2005). Antimicrobial Peptides: Pore Formers or Metabolic Inhibitors in Bacteria?. Nat. Rev. Microbiol..

[18] Zasloff M. (2002). Antimicrobial Peptides of Multicellular Organisms. Nature.

[19] Groß R., Bauer R., Krüger F., Rücker-Braun E., Olari L.-R., Ständker L., Preising N., Rodríguez A.A., Conzelmann C., Gerbl F. (2020). A Placenta Derived C-Terminal Fragment of β-Hemoglobin with Combined Antibacterial and Antiviral Activity. Front. Microbiol..

[20] Mahlapuu M., Håkansson J., Ringstad L., Björn C. (2016). Antimicrobial Peptides: An Emerging Category of Therapeutic Agents. Front. Cell. Infect. Microbiol..

[21] Wiegand I., Hilpert K., Hancock R.E.W. (2008). Agar and Broth Dilution Methods to Determine the Minimal Inhibitory Concentration (MIC) of Antimicrobial Substances. Nat. Protoc..

[22] Raghuraman H., Chattopadhyay A. (2007). Melittin: A Membrane-Active Peptide with Diverse Functions. Biosci. Rep..

[23] Dick G.W.A., Kitchen S.F., Haddow A.J. (1952). Zika Virus (I). Isolations and Serological Specificity. Trans. R. Soc. Trop. Med. Hyg..

[24] Papkalla A., Münch J., Otto C., Kirchhoff F. (2002). Nef Enhances Human Immunodeficiency Virus Type 1 Infectivity and Replication Independently of Viral Coreceptor Tropism. J. Virol..

