# Supplementary material for: Broad-Spectrum Antiviral and Antibacterial Activity of the Scorpion Venom Peptide HP1090
Source: Toxins (Basel). 2026 Jun 16;18(6):268. doi: 10.3390/toxins18060268 (PMC13307730; doi:10.3390/toxins18060268)
Supplement: Supplementary file 1 [file toxins-18-00268-s001.zip › toxins-4322235-supplementary.pdf]

Supplementary Materials: Broad-Spectrum Antiviral and Antibacterial Activity of the Scorpion Venom Peptide HP1090

Ariel J. Asuzano [1], Lia-Raluca Olari [1,2], Nourice Jaber [3], Verena Vogel [3], Marina S. Fam [4], Armando A. Rodríguez Alfonso [5,6], Nico Preising [5], Ludger Ständker [5], Barbara Spellerberg [3], Hans-Georg Breitinger [4], Ulrike Breitinger [4] and Jan Münch [1,*]

1 Institute of Molecular Virology, Ulm University Medical Center, 89081 Ulm, Germany; ariel.asuzano@uni-ulm.de (A.J.A.)
2 Department of Genomics, Medfuture Institute for Biomedical Research, Iuliu Hatieganu University of Medicine and Pharmacy, 400337 Cluj-Napoca, Romania
3 Institute of Medical Microbiology and Hygiene, Ulm University Medical Center, 89081 Ulm, Germany;
4 Department of Biochemistry, Faculty of Pharmacy and Biotechnology, German University in Cairo, Cairo 11835, Egypt
5 ULMTeC Core Facility for Functional Peptidomics, Ulm University Medical Center, 89081 Ulm, Germany
6 ULMTeC Core Facility of Mass Spectrometry and Proteomics, Ulm University Medical Center, 89081 Ulm, Germany
* Correspondence: jan.muench@uni-ulm.de

a

HP1090

Sequence: IFKAIWSGIKSLF
Length: 13
Terminal modifications: none
Formula: $C_{76}H_{116}N_{16}O_{16}$
Isoelectric point: 9.84
Average mass: 1509.8318 Da
Monoisotopic mass: 1508.8755 Da
Charge at pH=7.4 (cytoplasm): 1.9
GRAVY: 1.077
Abs 0.1% (=1 g/l) 3.643
%basic residues (K,R): 15.38
% Hydrophobic residues (A,V,L,I,M,F,W,Y,C,G): 69.23
% aromatic residues (F,W,Y) : 23.08

b

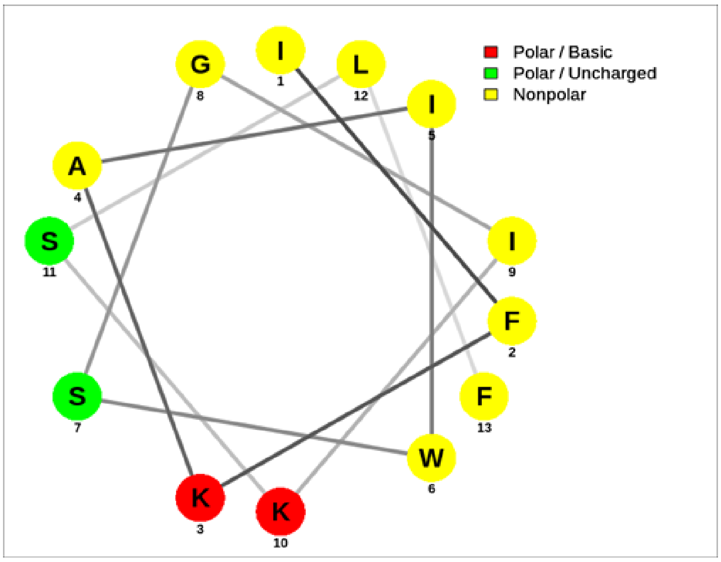

Supplementary Figure S1. Sequence and structural properties of HP1090. (a) Physicochemical properties of HP1090. **(b)** Helical wheel projection showing amphipathic distribution of hydrophobic (yellow), polar/uncharged (green), and polar/basic (red) residues.

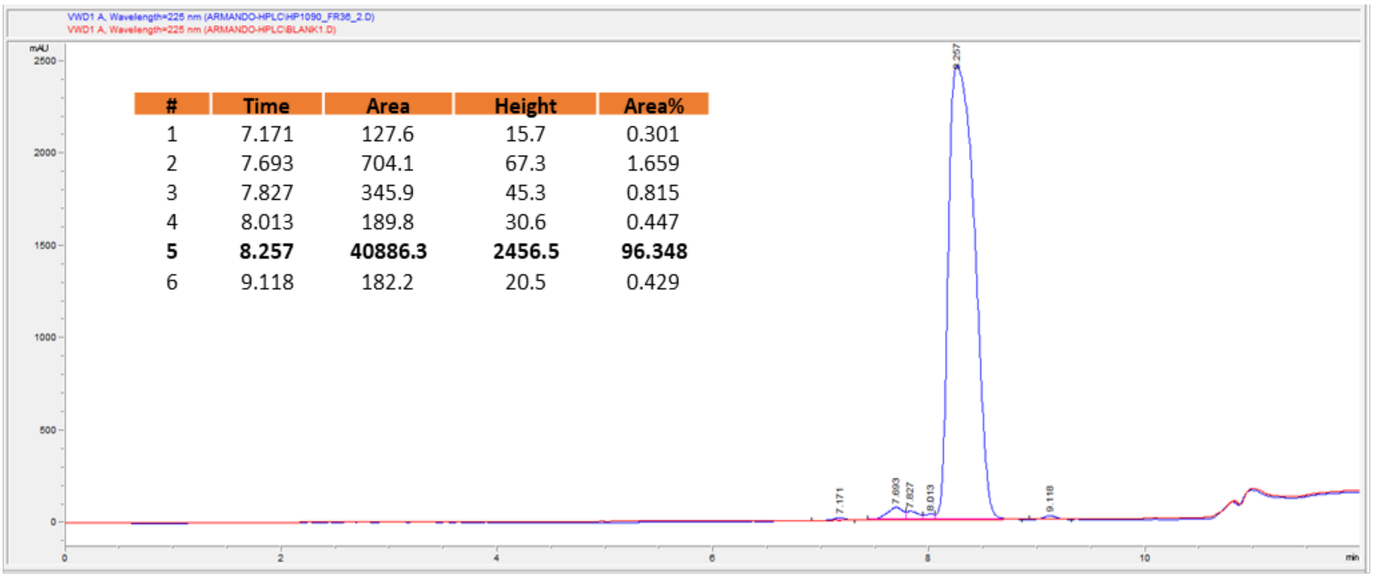

#	Time	Area	Height	Area%
1	7.171	127.6	15.7	0.301
2	7.693	704.1	67.3	1.659
3	7.827	345.9	45.3	0.815
4	8.013	189.8	30.6	0.447
**5**	**8.257**	**40886.3**	**2456.5**	**96.348**
6	9.118	182.2	20.5	0.429

Supplementary Figure S2. Reversed-phase chromatographic analysis of synthetic HP1090. Red trace: blank run. Blue trace: HP1090 run. Peak integration shows a purity of 96.348 %.

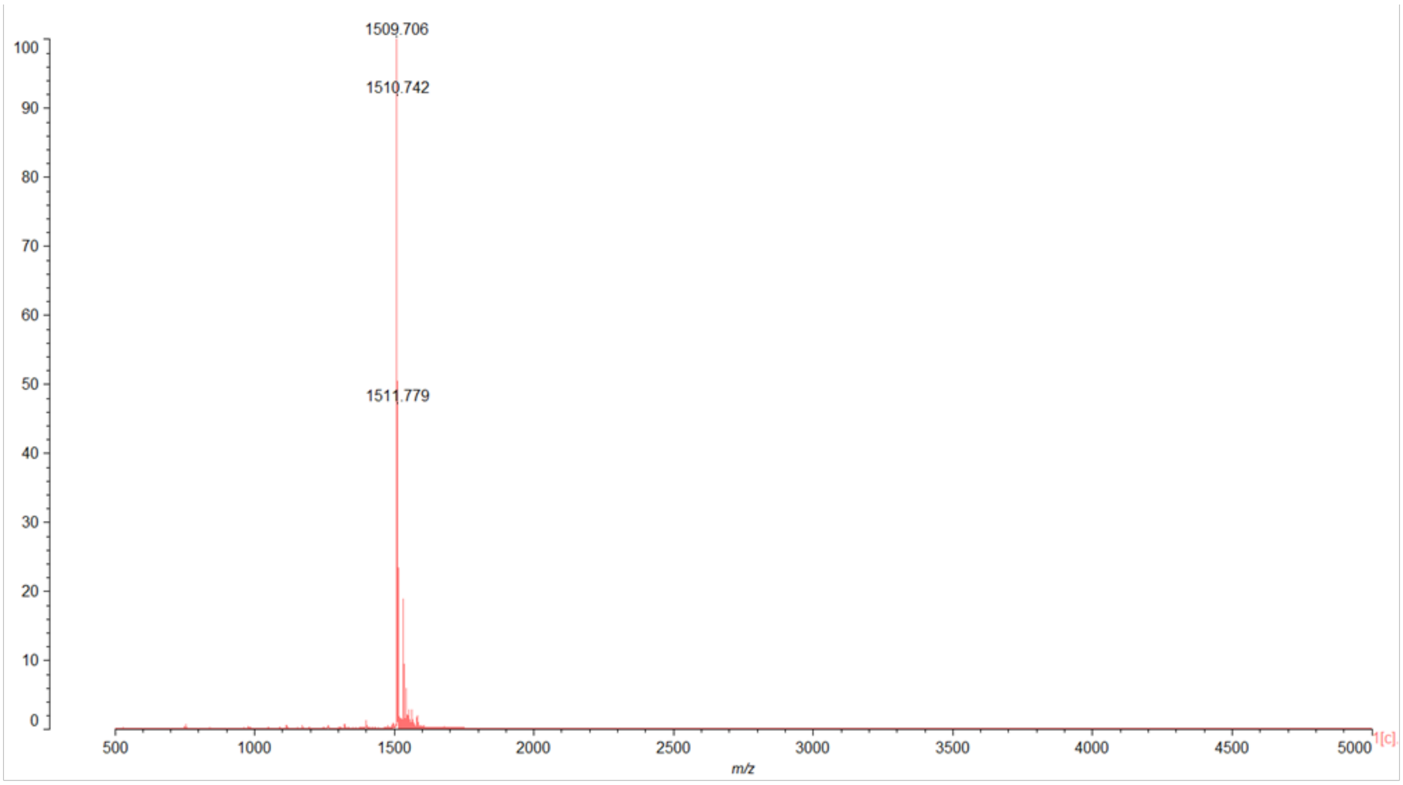

Supplementary Figure S3. MALDI-TOF mass spectrum of synthetic HP1090. The m/z value (1509.706) closely matches the theoretical one (1509.88).

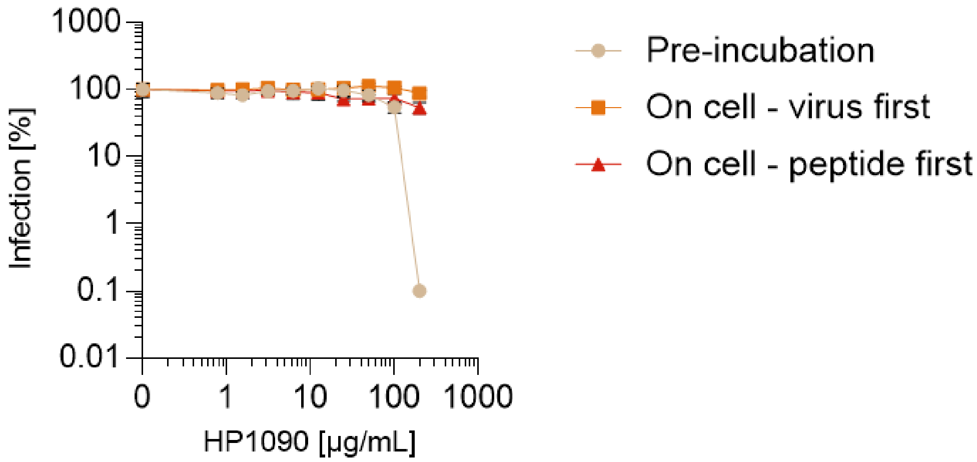

Supplementary Figure S4. Time-of-addition assay of HP1090 on ZIKV-infected Vero E6 cells. Three experimental configurations were used to assess the stage of HP1090 antiviral activity. Pre-incubation: HP1090 was incubated directly with ZIKV for 1 h at 37 °C prior to addition to Vero E6 cells. On cell—virus first: Vero E6 cells were infected with ZIKV for 1 h, followed by addition of HP1090. On cell—peptide first: HP1090 was added to Vero E6 cells for 1 h, cells were washed with PBS to remove peptide, and subsequently infected with ZIKV. All concentrations refer to on virus concentration (on-cell concentration is five-fold lower, similar to the pre-incubation experiment). Infection was quantified by a cell-based ZIKV immunodetection assay two days post-infection and normalized to untreated controls. Data represent mean ± SEM from a single experiment performed in triplicate.

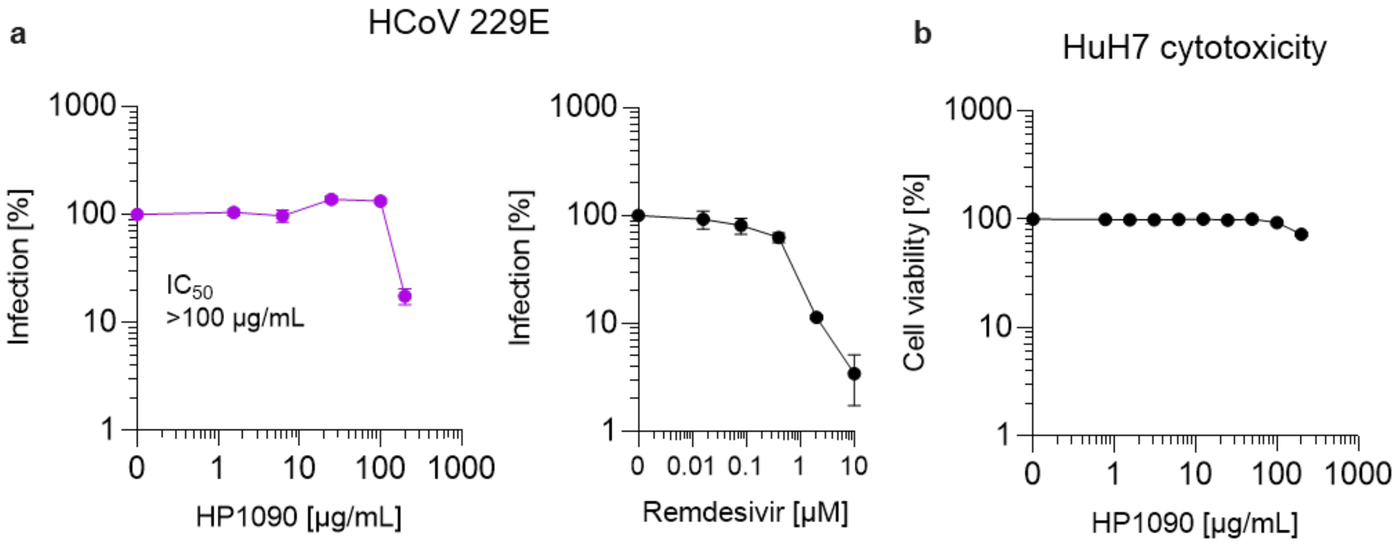

Supplementary Figure S5. Antiviral activity of HP1090 against human coronavirus 229E (HCoV-229E) and cytotoxicity on HuH7 cells. (**a**) HCoV-229E was pre-incubated with the indicated concentrations of HP1090 (left) or remdesivir (right) for 1 h at 33 °C prior to addition to HuH7 cells. Infection was quantified by cell-based immunodetection and normalized to untreated controls. IC_{50} >100 µg/mL. (**b**) Cytotoxicity of HP1090 on HuH7 cells assessed by MTT assay. Data represent mean ± SEM from three independent experiments, each performed in triplicate.
